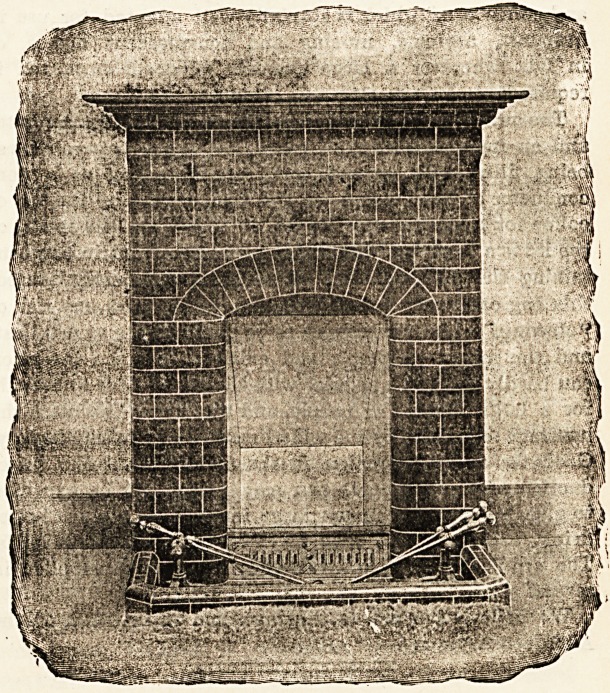# Practical Departments

**Published:** 1904-07-23

**Authors:** 


### PRACTICAL DEPARTMENTS.

### THE LANCASTER STOVE.

(MESSRS. BARNARD'S CATALOGUE.)

HEATING engineers seem to vie with one another in trying to produce artistically-designed and instructive catalogues. One of the best we have seen lately is that issued by Messrs. Barnard, Bishop, and Barnard, Limited, of Norwich. In the chapter upon fire grates the uninitiated are shown what one of the old (and if it must be said somewhat wasteful) fireplaces are like with its broad opening jack, and dogs, and we turn over the page containing this picturesque example with almost a regret that this is one of the cases where science has triumphed over art. Next we are shown some very instructive plans and sections, eight sets in all, and most of these are figures depicting how the firm's wares are built up. It would seem that the backs are made to slope either backwards or forwards to all useful angles between the obtuse and acute, and with or without vertical or horizontal bars. Some plans show deep recesses, some shallow, others convex, and others have rounded corners; in fact, were these designs not so well set out their number would be confusing. We do not think the racket regulator in the centre of the flue a good arrangement, it is not get-at-able; we fancy that the sweep would make short work of the fitting if it obstructed the free working of his brushes. We believe, however, it is probable that the firm is about to improve this system.

In the block we illustrate here it will be noticed that the mantel resembles built up brickwork, but this is not the case. It is formed of tiles upon an iron framework and can in consequence be quickly fixed up. With regard to the merits or demerits of the front from an artistic standpoint we will be silent, but certainly in this case "Art has concealed artifice." The name "Lancaster" refers to the design of the front only, which is perhaps a little severe in treatment, but we think it is very suitable to the class of building in which we are most interested. This front can be easily cleaned down, and we understand that practically any of Messrs. Barnard and Company's fire grates can be fitted to the design. The design of No. 903 in the catalogue ought to satisfy the section of the community who require art designs. By the way, we think that Messrs. Barnard's castings generally are very clean. Before we left the showroom in No. 110 Cannon Street we had an opportunity of examining some of the metal, also woodwork. Some of the designs for fenders and fire-irons are good, and a hardwood mantel commended itself to us especially. Coal boxes, kitchen ranges, boilers, and radiators, taps, gauges, are all included in this comprehensive catalogue; we hear that the cost of production of this work of art has been considerable and that copies are getting scarce.

### THE BALMORAL TEA URN.

Messrs. Marshall and Philip, of Union Street, Aberdeen, have brought out an excellent patent "combined tea and coffee urn," which is specially well adapted for use in hospitals, asylums, nursing and convalescent homes, or in fact wherever a large quantity of good warm tea is required.

The urn is made of glazed earthenware, and has an outer and an inner sheathing of metal, the space between these being filled with ground cork, which, acting as a non-conductor of heat, keeps up the temperature of the tea or coffee for a considerable length of time. On the inside of the lid is a hook on which may be hung a muslin bag containing the tea or coffee. When it is sufficiently infused, this bag can easily be removed, and so obviates any risk of "stewing." The advantages to be obtained are so manifest, and the means so simple and efficient, that the invention can hardly fail to be popular with those who have to provide for a large number of people, and who know how often the beverage obtained by the older methods is unsatisfactory. Good tea is often spoiled in the making, and the introduction of the Balmoral urn may reasonably be expected to give the patients in our large institutions a much better drink, and that, too, with no extra trouble to the officials. The urn is attractive in appearance, and can be supplied in several sizes.

We believe the urn is the invention of Dr. Reid, of the Aberdeen Asylum.

## Figures and Tables

**Figure f1:**